# *Col4a1* mutations cause progressive retinal neovascular defects and retinopathy

**DOI:** 10.1038/srep18602

**Published:** 2016-01-27

**Authors:** Marcel V. Alavi, Mao Mao, Bradley T. Pawlikowski, Manana Kvezereli, Jacque L. Duncan, Richard T. Libby, Simon W. M. John, Douglas B. Gould

**Affiliations:** 1 Department of Ophthalmology, School of Medicine, University of California, San Francisco, San Francisco, CA, 94143; 2 Department of Ophthalmology, University of Rochester, Rochester, NY, 14642; 3 Howard Hughes Medical Institute and The Jackson Laboratory, Bar Harbor, ME 04609; 4 Department of Anatomy and Institute for Human Genetics, School of Medicine, University of California, San Francisco, San Francisco, CA, 94143.

## Abstract

Mutations in collagen, type IV, alpha 1 (COL4A1), a major component of basement membranes, cause multisystem disorders in humans and mice. In the eye, these include anterior segment dysgenesis, optic nerve hypoplasia and retinal vascular tortuosity. Here we investigate the retinal pathology in mice carrying dominant-negative *Col4a1* mutations. To this end, we examined retinas longitudinally *in vivo* using fluorescein angiography, funduscopy and optical coherence tomography. We assessed retinal function by electroretinography and studied the retinal ultrastructural pathology. Retinal examinations revealed serous chorioretinopathy, retinal hemorrhages, fibrosis or signs of pathogenic angiogenesis with chorioretinal anastomosis in up to approximately 90% of *Col4a1* mutant eyes depending on age and the specific mutation. To identify the cell-type responsible for pathogenesis we generated a conditional *Col4a1* mutation and determined that primary vascular defects underlie *Col4a1*-associated retinopathy. We also found focal activation of Müller cells and increased expression of pro-angiogenic factors in retinas from *Col4a1*^+/Δ*ex41*^mice. Together, our findings suggest that patients with *COL4A1* and *COL4A2* mutations may be at elevated risk of retinal hemorrhages and that retinal examinations may be useful for identifying patients with *COL4A1* and *COL4A2* mutations who are also at elevated risk of hemorrhagic strokes.

Diseases involving abnormal proliferation of blood vessels in the retina, including diabetic retinopathy and age-related macular degeneration, are common causes of sudden and severe vision loss^1^. Like many diseases involving retinal neovascularization, newly formed blood vessels are prone to leak and rupture causing retinal detachments and hemorrhages leading to photoreceptor cell death and irreversible blindness.

Dominant-negative mutations in the genes encoding collagen, type IV, alpha 1 (COL4A1; OMIM: *120130) and alpha 2 (COL4A2; OMIM: *120090) are pleiotropic and contribute to a broad spectrum of disorders including myopathy, glaucoma and cerebrovascular disease^2^,^3^,^4^,^5^. Type IV collagens are extracellular matrix molecules that assemble into networks critical for basement membrane structure and function. There are six distinct isoforms (COL4A1–6) that are encoded by three pairs of orthologous genes^5^. COL4A1 and COL4A2 are present in nearly all basement membranes whereas COL4A3–6 are spatially restricted^6^,^7^. In the retina, COL4A1 and COL4A2 are mainly present in basement membranes of the vasculature and the choriocapillaries^8^,^9^. COL4A1 and COL4A2 assemble into heterotrimers in the endoplasmic reticulum when carboxy-terminal non-collagenous domains initiate heterotrimer formation. Heterotrimer assembly proceeds by winding along the triple helical domain that is defined by repeating Gly-Xaa-Yaa motifs^10^. Assembled and modified heterotrimers advance through the secretory pathway to the extracellular space where they polymerize into a network and interact with other extracellular matrix proteins to form basement membranes^5^,^11^,^12^.

*Col4a1* and *Col4a2* mutant mice model several types of human disease^2^,^3^,^13^,^14^,^15^. In order to better understand the consequences of *COL4A1* mutations in patients we characterized retinopathy in *Col4a1* mutant mice. We studied the *Col4a1*^Δ*ex41*^ mutation, which arises from a point mutation of a splice acceptor site preceding exon 41 resulting in exon 40 splicing directly to exon 42^2^,^5^. The transcript remains in frame and produces a protein that is missing 17 amino acids within the triple helical domain. *Col4a1*^+/Δ*ex41*^ mice on a C57BL/6J (B6) genetic background have relatively severe ocular pathology including cataracts, anterior segment dysgenesis, optic nerve hypoplasia, and glaucoma^13^,^16^. Dysgenesis and cataracts in *Col4a1*^+/Δ*ex41*^ mice on a B6 background make it difficult to image and study retinal phenotypes, however, these defects are genetically modified when crossed once (F1) to 129S6/SvEvTac (129) mice^13^,^16^. Most *Col4a1*^+/Δ*ex41*^ 129B6F1 mice have only mild anterior segment dysgenesis allowing visualization of the retinas *in vivo*. Here, we characterize progressive retinal pathologies using *in vivo* longitudinal imaging in *Col4a1*^+/Δ*ex41*^ 129B6F1 mice and correlate these findings with histopathological examinations. Moreover, we include retinal imaging of an allelic series of genetically matched *Col4a1* and *Col4a2* mutant mice. The allelic series comprised seven glycine mutations in the triple helical domain (six in COL4A1 and one in COL4A2) and one mutation in the COL4A1 non-collagenous (NC1) domain. Finally, we developed a conditional *Col4a1* mutation with which to genetically dissect differential cell-type contributions and identify the primary site of pathogenesis for retinopathy. By flanking exon 41 with *Lox P* sites we re-created the *Col4a1*^Δ*ex41*^ mutation in a cell-type specific manner. The data demonstrate that the *Col4a1* mutation causes highly penetrant and progressive retinopathy that is secondary to vascular defects. We propose that patients with *COL4A1* or *COL4A2* mutations may be at risk for developing vision-threatening retinopathy.

## Results

We have previously reported that *Col4a1*^Δ*ex41*^ causes ocular anterior segment dysgenesis on a B6 background that is suppressed in a 129B6F1 genetic context^13^. Therefore, we chose to study retinopathy in 129B6F1 mice to allow longitudinal retinal imaging *in vivo*. We imaged 332 eyes from 129B6F1 mice (140 *Col4a1*^+/+^ and 192 *Col4a1*^+/Δ*ex41*^) that ranged between three weeks to two years of age. In *Col4a1*^+/Δ*ex41*^ mice between postnatal day (P) 18 and P21, 6 out of 88 eyes had anterior segment dysgenesis, microphthalmia, corneal abrasions or lens opacities that precluded *in vivo* retinal imaging (Table 1). With age, more *Col4a1*^+/Δ*ex41*^ mice had anterior ocular complications (Table 1). Of the remaining 82 eyes, 44 had no observable retinal phenotype indicating that the penetrance of retinal pathology in *Col4a1*^+/Δ*ex41*^ 129B6F1 mice ranges between 36% and 43% by P21 (this range reflects the assumptions that eyes with anterior dysgenesis and that could not be imaged were either all unaffected or all affected, respectively). For *Col4a1*^+/Δ*ex41*^ mice older than one month the penetrance of retinal pathology ranged between 68% and 88%, suggesting that the lesions are progressive.

The most common type of *COL4A1* and *COL4A2* mutation in patients are missense mutations in conserved glycine residues of the Gly-Xaa-Yaa motifs in the triple helical domain^5^ and so we extended our investigation to an allelic series of mice with disease-relevant mutations. Seven of these mutations changed glycine residues (*Col4a1*^+/*G394V*^; *Col4a2*^+/*G646D*^; *Col4a1*^+/*G658D*^; *Col4a1*^+/*G912V*^; *Col4a1*^+/*G1038S*^; *Col4a1*^+/*G1180D*^; *Col4a1*^+/*G1344D*^) and one mutation changed an amino acid in the NC1 domain of COL4A1 (*Col4a1*^+/*S1582P*^)^15^,^17^,^18^. Indeed, all *Col4a1* and *Col4a2* mutant mouse lines showed retinal pathology that was similar to that observed in the larger cohorts of *Col4a1*^+/Δ*ex41*^ mice (see supplementary material, Figure S1 and Table S1).

**Figure 1:**
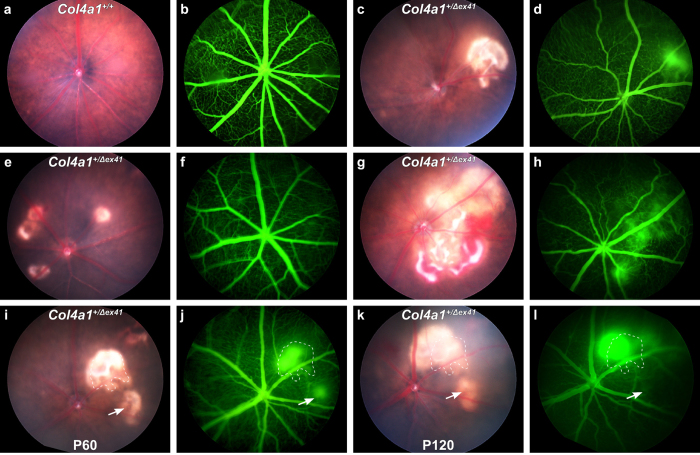
*Col4a1*^+/Δ*ex41*^ mice revealed variable retinal phenotypes when imaged *in vivo* by funduscopy, some of which associated with hyperfluorescent signals in fluorescein angiography (FA). Funduscopy (**a**) and FA (**b**) of *Col4a1*^+/+^ control mice were unremarkable. In contrast, *Col4a1*^+/Δ*ex41*^ mice (c–l), showed variable retinal phenotypes by funduscopy and a ramified and tortuous retinal vasculature by FA. One can distinguish between two types of lesions, larger yellow-white intraretinal lesions with irregular borders associated with hyperfluorescent signals in FA (**c**,**d**), as well as smaller disciform lesions with well-defined borders not associated with acute dye leakage (**e**,**f**). Occasionally we also found active sites of hemorrhage in *Col4a1*^+/Δ*ex41*^ mice (**g**,**h**). Longitudinal follow-up over two months from P60 to P120 of two lesions in the same eye of a *Col4a1*^+/Δ*ex41*^ mouse revealed that large hyperfluorescent lesions either persisted and grew in size (**i**–**l**, dotted area) or regressed into smaller, disciform lesions that no longer hyper-fluoresced on FA (**i**–**l**, arrow). Ages: (**a**–**f)**, 4 months; (**e**,**f**) 6 weeks; (**g**,**h**), 12 months.

Funduscopy and fluorescein angiography (FA) in *Col4a1*^+/+^ mice was unremarkable (Fig. 1a,b) with the exception of focal retinal atrophy in 2 out of 140 eyes (see supplementary material, Figure S2). Funduscopy in *Col4a1*^+/Δ*ex41*^ mice revealed two types of distinct intraretinal lesions that were often observed in different eyes of the same mouse and occasionally within the same eye. The first were yellow-white lesions that tended to be large with irregular borders (Fig. 1c) and the second were heterogeneous disciform lesions that tended to be small and white with well-defined borders (Fig. 1e). Occasionally, we identified eyes with vitreal hemorrhages and focally adherent posterior vitreous (Fig. 1g,h; see supplementary material, Figure S3 and supplemental 3D-reconstruction V1). All *Col4a1*^+/Δ*ex41*^ mice had abnormal vascular branching and vascular tortuosity detected by FA, however, the vessels did not reveal diffuse leakage that would have suggested gross compromise of the blood-retinal barrier. FA reinforced the distinction between classes of intraretinal lesions whereby a hyper-fluorescent signature accompanied the large, irregular lesions but not the small, disciform lesions (Fig. 1c,d compared to e and f).

We imaged *Col4a1*^+/Δ*ex41*^ eyes longitudinally for up to six months to follow progression of the pathology. In 6 out of 23 eyes, we identified the development of new lesions. Overall, small, defined lesions without hyper-fluorescence were stable, but some large hyper-fluorescent lesions changed and followed one of two fates. In 3 cases, the large hyper-fluorescent lesions persisted and grew in size. In 7 cases, these lesions regressed into small disciform lesions that no longer associated with a hyper-fluorescent signal on FA (Fig. 1i–l). These observations suggest that the large, diffuse lesions are early and dynamic and either progress with time or spontaneously resolve into small, defined lesions that are no longer permeable to fluorescein.

**Figure 2:**
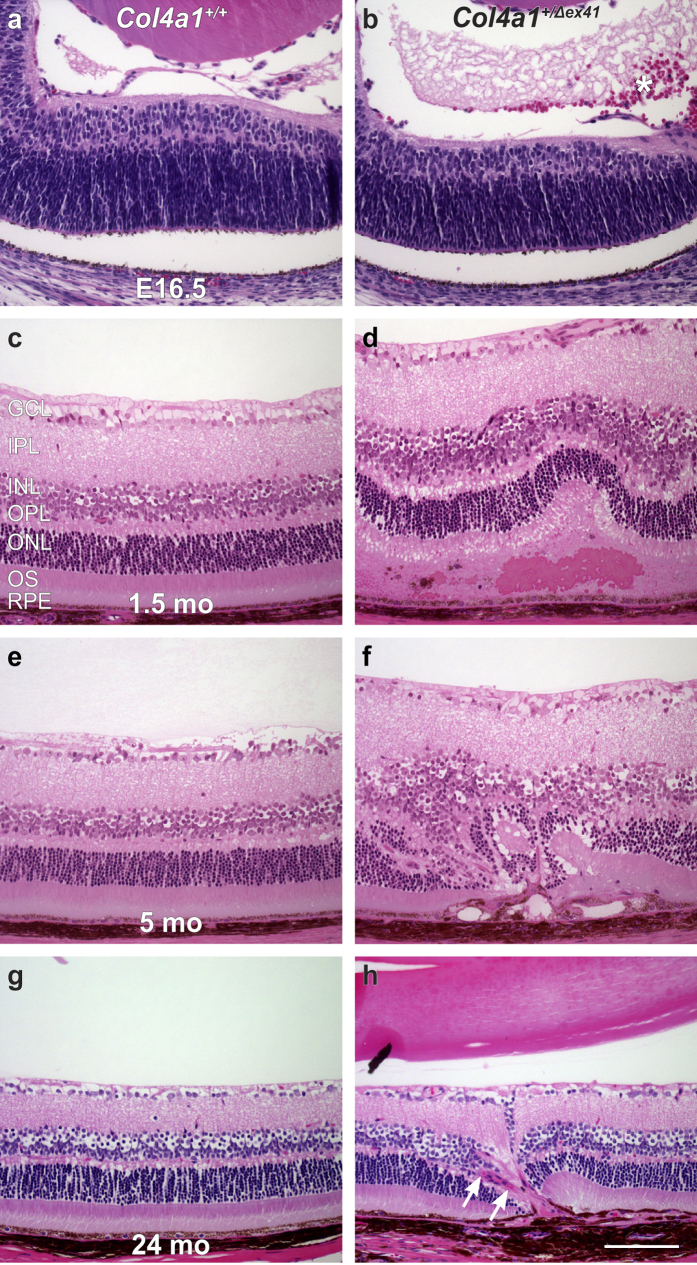
*Col4a1*^+/Δ*ex41*^ mice show numerous retinal abnormalities at different ages in light micrographs. Histological analysis of H&E stained retinal sections of *Col4a1*^+/+^ and *Col4a1*^+/Δ*ex41*^ mice at embryonic day (E) 16.5 (**a**,**b**), 1.5 months (**c**,**d**), 5 months (**e**,**f**) and 24 months (**g**,**h**). At younger ages, *Col4a1*^+/Δ*ex41*^ eyes predominantly show vitreal (**asterisk in b**) and subretinal hemorrhages (**d**). At older ages, *Col4a1*^+/Δ*ex41*^ eyes revealed the presence of abnormal blood vessels (**arrows in h**), fibrosis, and retinal restructuring (**f**,**h**). The histological findings were consistent with *in vivo* imaging studies. GCL, ganglion cell layer; IPL, inner plexiform layer; INL, inner nuclear layer; OPL, outer plexiform layer; ONL, outer nuclear layer; OS, outer segments; RPE, retinal pigment epithelium. Bar: 100 μm.

**Figure 3:**
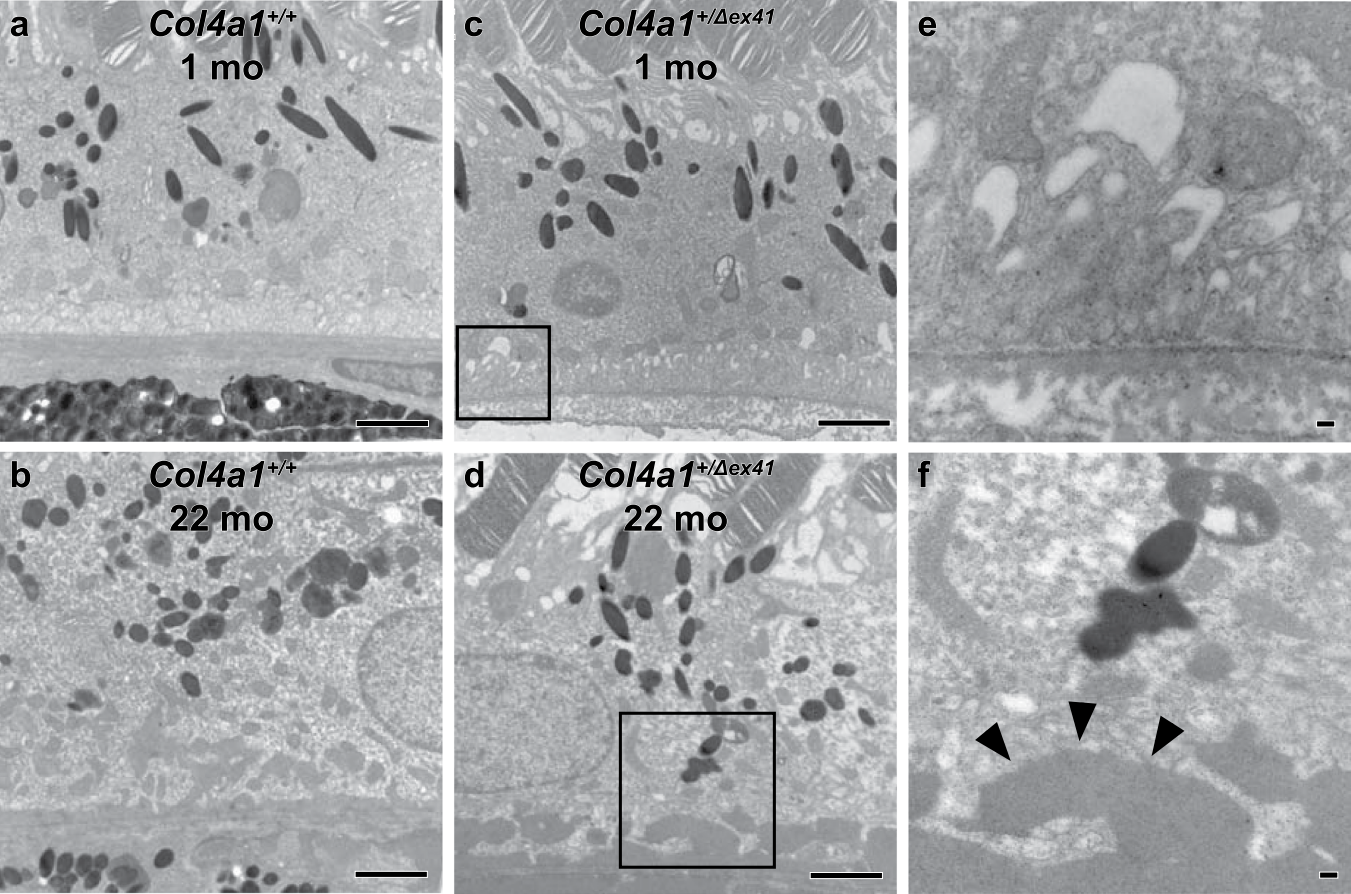
Ultra-structural analyses revealed age-dependent accumulation of amorphous deposits at the RPE-photoreceptor complex in *Col4a1*^+/Δex41^ mice. Electron micrographs of the photoreceptor/RPE/Bruch’s membrane complex revealed thickening of Bruch’s membrane compared to age-matched *Col4a1*^+/+^ littermate controls (**a**,**b**) in retinas from 1-month old *Col4a1*^+/Δ*ex41*^ mice (**c**,**e**). At 2 years of age, extensive accumulations of amorphous material were observed in *Col4a1*^+/Δ*ex41*^ mice (**d**,**f**, arrow heads) compared to *Col4a1*^+/+^ mice (**b**). (**e**,**f**) are higher magnifications of the insets in (**c**,**d**), respectively. Bars in (**a**–**c**,**e**): 2 μm; bar in (**e**): 100 nm; bar in (**f**): 50 nm.

To further understand the retinal pathology we analyzed more than 30 *Col4a1*^+/Δ*ex41*^ animals by light microscopy. We identified vitreous hemorrhages in *Col4a1*^+/Δ*ex41*^ mice as early as embryonic day (E) 16.5 that were not detected in *Col4a1*^+/+^ mice (Fig. 2a,b). Histological analyses of adult mice identified heterogeneous defects including subretinal hemorrhages, disrupted RPE and disorganized cellular and plexiform layers of the outer retina (Fig. 2c–h). Observations of retinal vascular pattern defects, hyperfluorescent signal of some intraretinal lesions and the apparent detection of abnormal blood vessels at the sites of pathogenic lesions are all consistent with the role for *Col4a1* in vascular disease. We labeled eyes from *Col4a1*^+/+^ and *Col4a1*^+/Δ*ex41*^ mice with antibodies against COL4A1 to detect vascular basement membranes. In *Col4a1*^+/+^ mice, labeling was appropriately restricted to the RPE-choriocapillaris complex and retinal blood vessels in the inner layers of the retina (see supplementary material, Figure S4a–c). In tissue sections through lesions in *Col4a1*^+/Δ*ex41*^ retinas we identified aberrant blood vessels among photoreceptors and adjacent to RPE cells (Figure S4d–i). We also examined the photoreceptor/RPE/choroid complex ultrastructurally using transmission electron microscopy. Again, eyes from *Col4a1*^+/+^ mice were structurally unremarkable showing only small amounts of amorphous accumulations within the RPE of aged animals (Fig. 3a,b). In contrast, *Col4a1*^+/Δ*ex41*^ mice showed thickened Bruch’s membrane with amorphous deposits and, in aged animals, large confluent deposits of amorphous material in the basal RPE (Fig. 3c–f). Together, the histologic and ultrastructural data demonstrate the presence of pathogenic blood vessels in the outer retina and changes in RPE cells and Bruch’s membrane. ERGs, however, revealed no significant differences between *Col4a1*^+/+^ and *Col4a1*^+/Δ*ex41*^ mice indicating that retinal function is otherwise normal and that focal lesions are not extensive enough to be detected on full field ERGs (see supplementary material, Figure S5).

**Figure 4:**
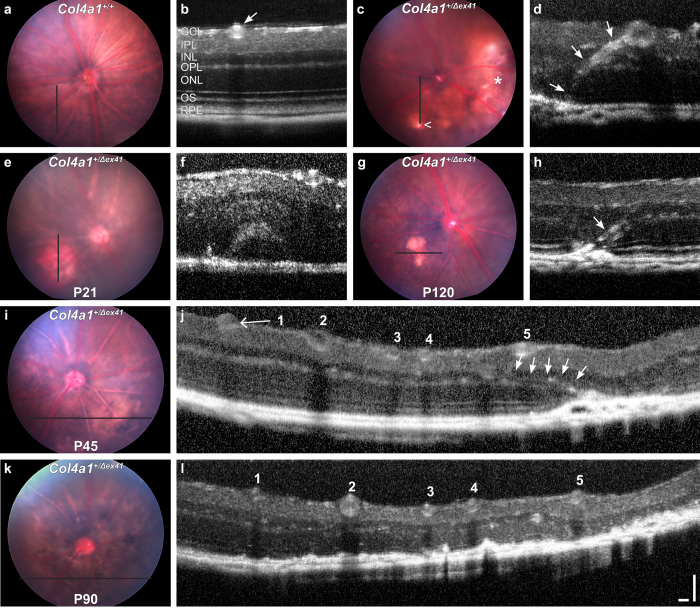
Image guided optical coherence tomography (OCT) reveals anastomosis of the retinal and choroidal vasculature and progressive retinopathy in eyes from *Col4a1*^+/Δ*ex41*^ animals. As exemplified for *Col4a1*^+/+^ eyes (**a**,**b**), image guided OCT can provide detailed *in vivo* information on retinal layers and blood vessels (arrow in b) at defined areas of the fundus (black line in **a**). We found in the inferior hemisphere of a *Col4a1*^+/Δ*ex41*^ eye a small and defined white lesions (**c**, arrow head), which showed chorioretinal anastomosis in OCT (**d**, arrows). A yellow-red lesion temporal in the same eye showed no anastomosis (not shown). A yellow-red lesion in the nasal-inferior hemisphere of an eye of a *Col4a1*^+/Δ*ex41*^ mouse at P21 (**e**) showed subretinal or sub-RPE fluid in OCT images (**f**). Follow-up at P120 showed that this lesion turned into a smaller disciform lesion (**g**) and OCT revealed chorioretinal anastomosis (**h**). A lesion with anastomosis in an eye of a P45 *Col4a1*^+/Δ*ex41*^ mouse (**I**,**j**) led to severe retinopathy with loss of most retinal layers at P90 (**k**,**l**). These data suggest that edema or hemorrhages from subretinal vessels precede vascular invasion into the retina and that neovascular events can spontaneously resolve or progress to widespread retinal degeneration. Ages: (**a**,**b**), 2 months; (**c**,**d**), 12 months. GCL, ganglion cell layer; IPL, inner plexiform layer; INL, inner nuclear layer; OPL, outer plexiform layer; ONL, outer nuclear layer; OS, outer segments; RPE, retinal pigment epithelium. Bars: 100 μm each.

To follow disease progression, we imaged *Col4a1*^+/+^ and *Col4a1*^+/Δ*ex41*^ eyes longitudinally with optical coherence tomography (OCT). OCT is an interferometric technique that provides detailed *in vivo* information on retinal layers that correlates well with *ex vivo* light microscopy^19^. In healthy retinas from *Col4a1*^+/+^ animals, OCT differentiates the retinal cellular and plexiform layers, and blood vessels appear as hyper-reflective structures (Fig. 4b, arrow). Examination of defined static lesions in *Col4a1*^+/Δ*ex41*^ mice revealed the presence of abnormal blood vessel remnants in the photoreceptor cell layer and outer segments (Fig. 4d, arrows; see supplementary material, Figure S4 and supplemental 3D-reconstructions V2 and V3). These lesions were strikingly similar in appearance to the abnormal vessels seen on histopathology. Longitudinal imaging of large, diffuse lesions demonstrated clear examples of disease progression. In one example from a young animal the original lesion had a red appearance on funduscopy – perhaps indicating retinal or subretinal hemorrhage (Fig. 4e,f). OCT did not detect abnormally localized blood vessels but showed an arcuate signal consistent with subretinal fluid. When this same lesion was imaged more than three months later it had the classic appearance of a small white static lesion on funduscopy and OCT detected RPE disruption and an obvious blood vessel in the outer retina that was not present earlier (Fig. 4g,h). In a second case we observed a large funduscopic lesion that was associated with RPE disruption and a blood vessel in the outer retina (Fig. 4I,j). When this same lesion was imaged 45 days later we observed loss of the outer retinal layers indicating progression of this lesion to retinal degeneration over the subretinal lesion (Fig. 4k,l). These data support a scenario whereby an initial insult (perhaps retinal or choroidal hemorrhages or edema) triggers abnormal invasion of blood vessels in the outer retina, whose permeability to sodium fluorescein indicates that they are leaky. The abnormal vessels either resolve leaving a disciform scar with chorioretinal anastomosis or continue to leak leading to progressively larger intraretinal lesions or even regional retinal degeneration with loss of the outer retinal layers over the vessels.

**Figure 5:**
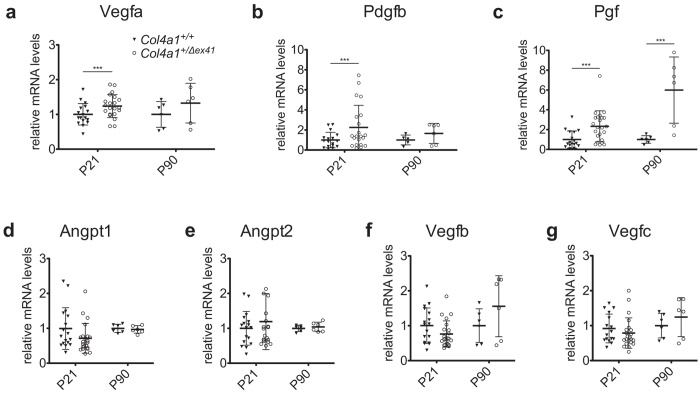
Elevated expression levels of pro-angiogenic factors in retinas of *Col4a1*^+/Δ*ex41*^ mice. We found significantly increased *Vegfa* (**a**) and *Pdgfb* expression (**b**) at P21 (*Col4a1*^+/+^: n = 17; *Col4a1*^+/Δ*ex41*^: n = 23) but not at P90 (n = 6 for both genotypes), while *Pgf* expression was significantly higher in *Col4a1*^+/Δ*ex41*^ retinas at both ages (**c**). We did not observe significant differences for *Vegfb*, *Vegfc*, *Angpt1* and *Angpt2* (**d–f**). Scatter plot with mean ± standard deviation; ***p < 0.05.

**Figure 6:**
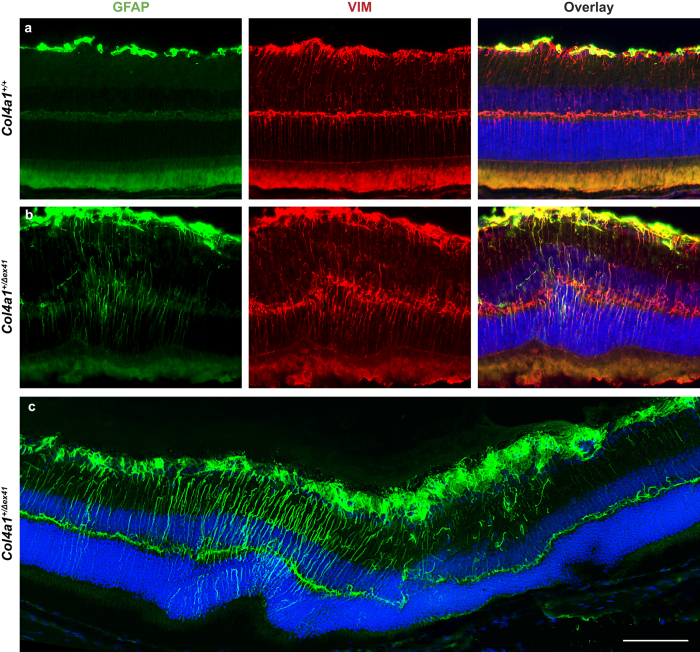
Focal Müller cell activation in retinas of *Col4a1*^+/Δ*ex41*^ mice. Retinal sections from *Col4a1*^+/Δ*ex41*^ mice showed prominent GFAP labeling associated with lesions, while adjacent areas or sections of eyes from *Col4a1*^+/Δ*ex41*^ mice without lesions showed only weak GFAP labeling (**a**). Vimentin co-labeling suggests that GFAP-positive cells are Müller cells (**b**). *Col4a1*^+/+^ mice did not show activation of Müller cells (**c**). Green, GFAP; red, Vimentin, Blue, DAPI nuclear staining. n = 5 eyes for each genotype. Bar: 100 μm.

Pro-angiogenic factors can contribute to retinal neovascularization^1^ and so we tested the expression levels of seven prominent angiogenic regulators. To this end, we harvested eyes from P21 and P90 *Col4a1*^+/Δ*ex41*^ mice and quantified expression levels of *Angpt1*, *Angpt2*, *Pdgfb, Pgf, Vegfa*, *Vegfb* and *Vegfc* by qPCR. At P21, we found that retinas from *Col4a1*^+/Δ*ex41*^ mice had significantly increased expression of *Vegfa, Pdgfb* and *Pgf* (*p* = 0.029, *p* = 0.032 and *p* = 0.003 respectively; Fig. 5) compared to *Col4a1*^+/+^ littermates. *Pgf* expression levels remained significantly elevated in *Col4a1*^+/Δ*ex41*^ retinas at P90 (*p* = 0.005; Fig. 5). *Angpt1*, *Angpt2, Vegfb* and *Vegfc*, did not have significant differences in expression levels at any age (Fig. 5). We have previously detected elevated levels of glial fibrillary acidic protein (GFAP) in cerebral cortices of *Col4a1*^+/Δ*ex41*^ mice^20^. GFAP labeling in the retina indicates activation of Müller cells, which are a major source of VEGF in models of ischemia-induced neovascularization and vascular leakage^21^. Consistent with these observations we identified increased GFAP labeling associated with lesions in retinas from *Col4a1*^+/Δ*ex41*^ mice but not in other areas from the same eye or in eyes from *Col4a1*^+/+^ mice (Fig. 6).

**Figure 7:**
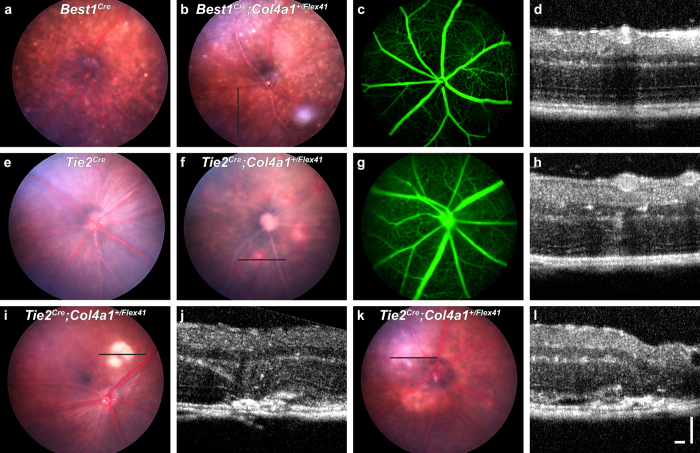
Conditional expression of mutant COL4A1 indicates that primary vascular insults underlie retinopathy. *Best1*^*Cre*^*;Col4a1*^+/*Flex41*^mice, which conditionally express mutant collagen in RPE, did not show pathological changes in the fundus even when aged to one year (**a–d**). Note: The different appearance of the fundus in both mouse lines (**a**,**b**) was associated with the *Best1*^*Cre*^ and has been described previously. Funduscopy of *Tie2*^*Cre*^ mice (**e**) was indistinguishable from *Col4a1*^+/+^ mice. *Tie2*^*Cre*^*;Col4a1*^+/*Flex41*^ mice conditionally express mutant collagen in vascular endothelial cells (**f–l**) and displayed all of the retinal phenotypes seen in *Col4a1*^+/Δ*ex41*^ animals, including subretinal or sub-RPE fluid (**f–h**), disciform scar formation with anastomosis (**i**,**j**), and regional retinal atrophy (**k**,**l**). FA also revealed ramified and tortuous retinal vasculature (**g**). These data demonstrate that vascular endothelial cells account for the retinal phenotype observed in *Col4a1*^+/Δ*ex41*^ mice. Ages: (**f–h**), 3 weeks; (**i**,**j**), 4 months; (**k**,**l**), 5 months. Bars: 100 μm each.

Intraretinal lesions are associated with disrupted RPE and we identified ultra-structural RPE changes. It is possible that primary RPE defects trigger pathogenic blood vessel invasion. Alternatively, *Col4a1* mutation is well established to cause vascular defects and blood vessels may be the primary site of pathogenesis. To identify the primary site of pathogenesis we generated a conditional *Col4a1* mutation^15^. We designed a construct whereby *LoxP* sites flanked exon 41 (called *Col4a1*^*Flex41*^), which allows generation of the *Col4a1*^Δ*ex41*^ mutation in a cell-type specific manner (supplemental material, Figure S6). We crossed *Col4a1*^+/*Flex41*^ mice to *Best1*^*Cre*^ or *Tie2*^*Cre*^ mice to drive expression of mutant *Col4a1* in RPE or vascular endothelial cells, respectively^22^,^23^. The *Col4a1*^*Flex41*^ allele and Cre lines were all on a C57BL/6J background and so the following experiments were done in this genetic context. First, we confirmed the appropriate expression of the Cre lines by funduscopy of mice that expressed Cre and the TdTomato fluorescent reporter and validated recombination of the conditional allele using reverse transcription PCR of retinal tissue (Figure S6e–j). We then examined the conditionally mutant offspring and control littermates by funduscopy, FA and OCT. *Best1*^*Cre*^ mice had small, white retinal spots on funduscopy irrespective of the presence or absence of the conditional allele (Fig. 7a,b). These do not phenocopy the *Col4al*^+/Δ*ex41*^ pathology and have been reported previously^24^. RPE-specific expression of mutant *Col4a1* did not cause pathogenic changes (n = 50 eyes) even when mice were aged for over one year (Fig. 7b–d). In contrast to control mice (*Col4a1*^+/+^*; Tie2*^*Cre*^ and *Col4a1*^+/*Flex41*^mice that did not carry the Cre transgene), the *Tie2*^*Cre*^;*Col4a1*^+/*Flex41*^ progeny had retinal vascular tortuosity and intraretinal lesions that resembled *Col4a1*^+/Δ*ex41*^ eyes (Fig. 7f–l, supplemental 3D-reconstructions V4). These data demonstrate that vascular defects are the primary cause underlying *Col4a1*-retinopathy.

## Discussion

Here we report spontaneous retinal and subretinal neovascular lesions in *Col4a1* mutant mice. Longitudinal fundus, FA and OCT analyses suggest that edema from leakage or hemorrhages of the retinal or choroidal vasculature precedes retinal neovascularization and that new vessels can eventually form retinal-choroidal anastomoses. Newly formed retinal vessels are permeable to sodium fluorescein and can spontaneously resolve leaving subretinal scars or may persist. Persistent vessels that continue to show hyperfluorescent signal on FA were associated with progressive lesions with advancing loss of photoreceptors. Conditional expression of mutant *Col4a1* in vascular endothelium, but not RPE, phenocopies the retinal defects in *Col4a1*^+/Δ*ex41*^ mice indicating that pathology is driven by primary vascular insults. Expression levels of *Vegfa, Pdgfb* and *Pgf* were significantly elevated in retinas with neovascularization and vascular lesions were locally associated with increased GFAP – a marker for Müller cell activation.

Most of the vascular lesions that we observe in *Col4a1*^+/Δ*ex41*^ mice resemble retinal angiomatous proliferation (RAP). RAP is a particular sub-type of pathologic retinal neovascularization^25^,^26^. In contrast to choroidal neovascularization (CNV) typically observed in wet AMD, RAP lesions originate in the retinal vasculature. However, they share a common outcome of high risk for sudden and debilitating vision loss^27^. In addition to occurring in a sub-set of AMD patients, RAP lesions are prevalent in Coats’ Disease^28^ and Familial Exudative Vitreoretinopahty (FEVR)^29^. RAP is proposed to progress through three stages; 1) formation of intraretinal neovascularization arising from the deep capillary vascular plexus, 2) growth of these abnormal retinal vessels toward and into the subretinal space and 3) anastomosis of retinal vessels and the choroidal vascular system^26^,^30^.

There are two well-characterized genetic models of spontaneous RAP, NRV2 mice^31^ and *Vldlr*^*−/−*^ mice^32^,^33^. Both models exhibit the three stages of progression starting with spontaneous neovascularization arising from the deep internal vascular plexus and extending toward the RPE where they eventually form chorioretinal anastomoses. Both models are also associated with activated Müller cells and elevated VEGF is also seen in other models of retinal neovascularization^33^,^34^,^35^,^36^. Accordingly anti-VEGF therapies were able to reduce neovascularization, at least temporarily, and might represent an available therapy to prevent vision loss in patients with RAP^31^,^33^,^34^. However, it is unclear the extent to which VEGF expression reflects a shared underlying mechanism or distinct tributary mechanisms that converge to this common outcome. The gene and mechanism underlying recessive NRV2 is still unknown. *Vldlr* is expressed in multiple tissues including the vascular endothelial and smooth muscle cells^37^. There is evidence suggesting that loss of VLDLR may cause RAP by abnormal de-repression of WNT signaling, which leads to increased *Vegf* expression^36^. This hypothesis is supported by the fact that mutations in other members of the WNT signaling pathway cause retinal vascular proliferative diseases including FEVR and Norrie disease^35^,^38^.

In contrast to NRV2 and *Vldlr*^*−/−*^ models, neovascular lesions and chorioretinal anastomosis in *Col4a1* mutant mice were less synchronous with later onset and were more focal with only a few lesions per eye. Additionally, the origin of many abnormal vessels in *Col4a1* mutant mice appears to be in the superficial vascular plexus compared to the deep vascular plexus in the NRV2 and *Vldlr*^*−/−*^ models (Fig. 4d and Figure S6). The cause of increased expression levels of pro-angiogenic factors in *Col4a1* mice is presently unknown and multiple possibilities exist. For example, Arresten, a proteolytic fragment of COL4A1 has anti-angiogenic properties and regulates *Vegf* via integrin-α1β1 signaling^39^,^40^. Reduced secretion of mutant COL4A1 could diminish extracellular Arresten leading to increased *Vegf* expression. Alternatively, edema resulting from abnormal blood vessels could create a hypoxic environment, which can promote *Vegf* and *Pgf* expression^41^,^42^,^43^. VEGF and PGF act synergistically to promote angiogenesis in pathological conditions^44^, while they play distinct roles during retinal development^45^. While we show an association of angiogenic factors with neovascular lesions we cannot conclude if the angiogenic factors are primary or secondary to new blood vessel formation. The low frequency and asynchronicity of the neovascular lesions make dissecting causation difficult.

Whether the upstream pathways are shared or not, RAP pathogenesis appears to be uniformly mediated by abnormally elevated expression of *Vegf*. However, the cellular source of VEGF remains unknown. Each of the mouse models shows an association of retinal neovascularization with Müller cell activation, which could be a source of VEGF^21^. However, neovascularization appears to be directional toward the subretinal space, implying that the VEGF source is in the outer retina. Infiltrating macrophages are present in the subretinal space at the sites of lesions and their depletion can reduce CNV lesion size suggesting that they might be a source of VEGF^34^,^41^,^46^,^47^. Alternatively, perturbations of directional VEGF secretion from the polarized RPE may result in apical VEGF secretion that attracts neovascularization into the subretinal space^36^. Identifying the cellular source of VEGF up-regulation will be important both for understanding the contributing pathways and for treating the disease. RAP responds well to anti-VEGF therapies; however targeting the primary insults instead of, or in addition to, VEGF up-regulation might prove to be more efficacious in preventing RAP onset or progression.

Mutations in *COL4A3*, *COL4A4* and *COL4A5* cause Alport Syndrome for which the main consequence is renal failure^48^. Patients with Alport Syndrome have perimacular ‘dot and fleck retinopathy’ which is ascribed to abnormalities in the inner limiting membrane/nerve fiber layer^49^ and that does not affect vision. Here we show that, in addition to increased risk for cerebrovascular disease (and other multi-systemic complications), patients with *COL4A1* or *COL4A2* mutations should be carefully monitored for progressive retinal neovascularization that could lead to sudden blindness. Indeed, patients with *COL4A1* mutations show early retinal pathologies including retinal hemorrhages and vascular tortuosity^50^,^51^. Interestingly, PGF can lead to vascular tortuosity, aneurysm and increased vascular spouting in the context of diabetic retinopathy^52^. Retinal tortuosity and RAP may be useful markers with which to identify patients with *COL4A1* and *COL4A2* mutations and who are also at elevated risk of hemorrhagic strokes^53^. The extent to which retinal and cerebral vascular defects caused by *COL4A1* mutation are shared remains to be determined. However, further understanding of retinal vascular pathology could be useful in developing therapeutics to prevent hemorrhagic strokes in patients with *COL4A1* and *COL4A2* mutations.

## Materials & Methods

### Animals

*Col4a1*^+/Δ*ex41*^ mice have a point mutation in the splice acceptor site of *Col4a1* exon 41 (NM_009931.2: c.3506-1G>A; NCBI Release 38.1). The mutation and ocular phenotypes were originally identified during a mutagenesis screen conducted at The Jackson Laboratory and were described previously^2^,^3^,^13^,^20^. *Col4a1* and *Col4a2* missense mutant mice were described previously^15^,^17^,^18^. Each strain was iteratively crossed to C57BL/6J mice for at least five generations and subsequently crossed to 129S6/SvEvTac mice to generate *Col4a1* and *Col4a2* mutant mice on a 129B6F1 genetic background. *Col4a1*^+/Δ*ex41*^ animals were F1 progeny from *Col4a1*^+/Δ*ex41*^ C57BL/6J (N>20 generations) and 129S6/SvEvTac mice. All findings were compared to *Col4a1*^+/+^ littermate controls. inGenious Targeting Laboratory (iTL; Stoney Brook, NY, USA) generated a conditional *Col4a1* mutant allele by flanking exon 41 with *Lox P* sites (called *Col4a1*^*Flex41*^). The construct (supplemental materials, Figure S5a,b) was generated from a C57BL/6 BAC sub-cloned into pSP72 (Promega; Madison, WI, USA). The targeting vector (10ug) was linearized and transfected by electroporation of iTL BA1 (C57BL/6 × 129/SvEv) hybrid embryonic stem (ES) cells. ES cell clones were expanded, successful targeting was confirmed by PCR and then microinjected into C57BL/6 blastocysts. Resulting chimeras were mated to C57BL/6 mice to generate heterozygous offspring. Tail DNA from the progeny was tested for the presence of both *Lox P* sites by PCR and sequencing. Positive mice were shipped to UCSF and iteratively backcrossed to C57BL/6J mice for at least 10 generations. *Col4a1*^+/*Flex41*^ C57BL/6J mice were crossed to Tg(Best1-Cre)1Jdun C57BL/6J^22^ or Tg(Tie2-Cre)1Rwng C57BL/6J^23^ mice for conditional mutant expression in retinal pigmented epithelium or vascular endothelial cells, respectively. We tested for successful deletion of *Col4a1* exon 41 in conditional mice with primers exon_41-F and exon_41-R, and recombination specific primers exon_41_RC-F and exon_41_RC-R. For a list of primers see supplemental materials. All animals were maintained in full-barrier facilities free of specific pathogens on a 12-hour light/dark cycle with food and water *ad libitum*. All experiments were compliant with the ARVO Statement for the Use of Animals in Ophthalmic and Vision Research and approved by the Institutional Animal Care and Use Committee at University of California, San Francisco.

### Funduscopy, Angiography, and OCT

Mice were anesthetized with a steady flow of 1.5 to 3% isofluorane. The eyes were topically anesthetized with one drop of proparacaine, dilated with one drop of a 1:1 mixture of 1% tropicamide and 2.5% phenylephrine. The corneas were kept moist with regular application of 2.5% methylcellulose. Eyes were examined using the Micron III retinal imaging system (Phoenix Research Labs, CA, USA) and raw images were adjusted for levels, enhanced contrast, and sharpened by applying an unsharp mask (100%, 2px, 0) using Photoshop CS6 (Adobe, Ca, USA). For angiography, animals received an intraperitoneal injection of 10 mg/ml fluorescein in PBS at a dose of 10 μl/g body weight. After 1 minute, eyes were imaged with the Micron III using the GFP-A-Basic-000 filter set (Semrock, NY, USA). Spectral domain OCT images were acquired with the Micron Image Guided SD-OCT System (Phoenix Research Labs) by averaging 10 to 20 scans. Levels were adjusted using Photoshop CS6 (Adobe).

### Electroretinography

Mice were dark-adapted overnight and anesthetized with intraperitoneal injection of ketamine (15 mg/g body weight) and xylazine (7 mg/g), and body temperature was maintained constant using a heated water pad. Gold electrodes were placed on the corneal surface of dilated eyes (1% atropine sulfate) and referenced to a gold wire placed in the mouth. A needle electrode inserted in the tail served as ground. A drop of 2.5% methylcellulose ensured electrical contact and corneal integrity. Signals were amplified (×10 000, CP511 AC amplifier, Grass Instruments, RI, USA), sampled every 0.8 ms, and averaged. Scotopic electroretinograms (ERGs) were recorded upon presenting short-wavelength flashes in a Ganzfeld dome, which varied over a 4.0 log unit range of intensities up to the maximum allowable by the photopic stimulator (PS33 Plus, Grass Technologies, RI, USA). Photopic ERGs were recorded with white flashes on a rod-saturating background after 10 min of light adaptation. Responses were computer averaged at all intensities with up to 50 records averaged for the weakest signals. A signal rejection window was used to eliminate electrical artifacts produced by blinking and eye movements.

### Histology and Electron Microscopy

Mice were euthanized, the superior pole of each eye marked before enucleation and immediately immersed in cold fixative (1% paraformaldehyde, 2% glutaraldehyde, and 0.1 M cacodylate buffer) for 24 hours, after which they were transferred to cold 0.1 M cacodylate buffer solution for an additional 24 hours. Samples were embedded in methacrylate historesin, and sections were cut and stained with hematoxylin and eosin (H&E). The eyes for ultrastructural studies were fixed in one-half strength Karnovsky fixative (2% formaldehyde and 2.5% glutaraldehyde in 100 mM cacodylate buffer, pH 7.4, containing 0.025% CaCl2) for at least 24 hours. Eyes were cut into small wedges, rinsed in 100 mM cacodylate buffer (pH 7.4), and post-fixed with 1% osmium tetroxide for 2 hours. Tissues from all four quadrants of each eye were subsequently processed for transmission electron microscopy.

### Immunohistochemistry

Eyes were fixed in 4% paraformaldehyde for 1 hour, cryo-protected in 30% sucrose in PBS and embedded in optimal cutting temperature compound (Sakura Finetek, CA, USA). 10 μM sections were permeabilized for 10 min in 0.1% Triton X-100 in PBS and incubated for 60 min in M.O.M. blocking reagent (Vector Inc., CA, USA). Sections were immunolabeled at 4 °C over night with goat anti-type IV collagen (1:10, Southern Biotech, AL, USA) or rabbit polyclonal anti-GFAP antibody (1:1000, Dako, Glostrup, Denmark) diluted in M.O.M. blocking reagent (Vector Laboratories, CA, USA) and incubated with corresponding secondary antibodies for 60 min. Sections were mounted in Mowiol containing 100 ng/ml DAPI (Calbiochem, NJ, USA).

### Microscopy

All images were taken on a Zeiss AxioImager M1 microscope (Zeiss, NY, USA) with an AxioCam MRm or AxioCam ICc3 digital camera with the AxioVision software 4.6. Post-acquisition images were processed using Photoshop CS6 (Adobe).

### Molecular Biology

RNA was isolated using the Direct-zol RNA MiniPrep kit (Zymo Research, CA, USA) from retinas homogenized in TRIzol Reagent (Life Technologies, CA, USA) by passing through 29-gauge needle. Quantity and purity of total RNA was assessed by Nanodrop (Thermo Fisher Scientific, DE, USA) and 500 ng of RNA was reverse transcribed using iScript Reverse Transcriptase Supermix (Biorad, CA, USA). Quantitative PCR was performed on 1 ng of cDNA with 1 μM of each primer and the SsoAdvanced SYBR Green Supermix (Biorad) in a two-step cycling protocol (denaturing: 5 s at 95 °C; annealing/extension: 30 s at 60 °C; 40 cycles) on a BioRad CFX96 and normalized to *Tbp* (NM_013684)^54^. For a list of primers see.

### Statistical analysis

For qPCR, all data were compared by Student two-tailed *t-*tests and differences were considered statistically significant for *p-*values below 0.05.

## Additional Information

**How to cite this article**: Alavi, M. V. *et al.* *Col4a1* mutations cause progressive retinal neovascular defects and retinopathy. *Sci. Rep.* **6**, 18602; doi: 10.1038/srep18602 (2016).

## Supplementary Material

Supplementary Information

Supplementary Video S1

Supplementary Video S2

Supplementary Video S3

Supplementary Video S4

## Figures and Tables

**Table 1:** Frequency of ocular findings in *Col4a1* ^*+/Δex41*^ 129B6F1 mice.

Age	Sample size (eyes)	Anterior dysgenesis	Unremarkable retina	Irregular lesions	Defined lesions
P18-P21	88	6	44	31	8
1–2 mo	26	2	9	5	10
3–6 mo	34	3	6	11	16
10–3 mo	32	7	6	15	4
>15 mo	12	8	0	4	0
total	192	26	63	66	38

## References

[b1] Campochiaro P. A. Ocular neovascularization. J Mol Med (Berl) 91, 311–321 (2013). 23329331 10.1007/s00109-013-0993-5 PMC3584193

[b2] Gould D. B. *et al.* Mutations in Col4a1 cause perinatal cerebral hemorrhage and porencephaly. Science 308, 1167–1171 (2005). 15905400 10.1126/science.1109418

[b3] Gould D. B. *et al.* Role of COL4A1 in small-vessel disease and hemorrhagic stroke. N Engl J Med 354, 1489–1496 (2006). 16598045 10.1056/NEJMoa053727

[b4] Plaisier E. *et al.* COL4A1 mutations and hereditary angiopathy, nephropathy, aneurysms, and muscle cramps. N Engl J Med 357, 2687–2695 (2007). 18160688 10.1056/NEJMoa071906

[b5] Kuo D. S., Labelle-Dumais C. & Gould D. B. COL4A1 and COL4A2 mutations and disease: insights into pathogenic mechanisms and potential therapeutic targets. Hum Mol Genet 21, R97–110 (2012). 22914737 10.1093/hmg/dds346 PMC3459649

[b6] Sado Y. *et al.* Establishment by the rat lymph node method of epitope-defined monoclonal antibodies recognizing the six different alpha chains of human type IV collagen. Histochem Cell Biol 104, 267–275 (1995). 8548560 10.1007/BF01464322

[b7] Uechi G., Sun Z., Schreiber E. M., Halfter W. & Balasubramani M. Proteomic View of Basement Membranes from Human Retinal Blood Vessels, Inner Limiting Membranes, and Lens Capsules. J Proteome Res 13, 3693–3705 (2014). 10.1021/pr5002065 24990792

[b8] Chen L., Miyamura N., Ninomiya Y. & Handa J. T. Distribution of the collagen IV isoforms in human Bruch’s membrane. Br J Ophthalmol 87, 212–215 (2003). 10.1136/bjo.87.2.212 PMC1771521 12543754

[b9] Bai X., Dilworth D. J., Weng Y. C. & Gould D. B. Developmental distribution of collagen IV isoforms and relevance to ocular diseases. Matrix Biol 28, 194–201 (2009). 19275937 10.1016/j.matbio.2009.02.004 PMC4749355

[b10] Khoshnoodi J., Cartailler J. P., Alvares K., Veis A. & Hudson B. G. Molecular recognition in the assembly of collagens: terminal noncollagenous domains are key recognition modules in the formation of triple helical protomers. J Biol Chem 281, 38117–38121 (2006). 17082192 10.1074/jbc.R600025200

[b11] Tsilibary E. C. & Charonis A. S. The role of the main noncollagenous domain (NC1) in type IV collagen self-assembly. J Cell Biol 103, 2467–2473 (1986). 3782304 10.1083/jcb.103.6.2467 PMC2114606

[b12] Mao M., Alavi M. V., Labelle-Dumais C. & Gould D. B. Type IV Collagens and Basement Membrane Diseases: Cell Biology and Pathogenic Mechanisms. in Current Topics in Membranes, Vol. 76, 61-116 (2015). 26610912 10.1016/bs.ctm.2015.09.002

[b13] Gould D. B., Marchant J. K., Savinova O. V., Smith R. S. & John S. W. Col4a1 mutation causes endoplasmic reticulum stress and genetically modifiable ocular dysgenesis. Hum Mol Genet 16, 798–807 (2007). 17317786 10.1093/hmg/ddm024

[b14] Van Agtmael T. *et al.* Dominant mutations of Col4a1 result in basement membrane defects which lead to anterior segment dysgenesis and glomerulopathy. Hum Mol Genet 14, 3161–3168 (2005). 16159887 10.1093/hmg/ddi348

[b15] Jeanne M., Jorgensen J. & Gould D. B. Molecular and Genetic Analyses of Collagen Type IV Mutant Mouse Models of Spontaneous Intracerebral Hemorrhage Identify Mechanisms for Stroke Prevention. Circulation 131, 1555–1565 (2015). 25753534 10.1161/CIRCULATIONAHA.114.013395 PMC4497509

[b16] Mao M. *et al.* Strain Dependent Anterior Segment Dysgenesis and Progression to Glaucoma in Col4a1 Mutant Mice. Invest Ophthalmol Vis Sci 56, 6823-6831 (2015). 26567795 10.1167/iovs.15-17527 PMC4627250

[b17] Favor J. *et al.* Type IV procollagen missense mutations associated with defects of the eye, vascular stability, the brain, kidney function and embryonic or postnatal viability in the mouse, Mus musculus: an extension of the Col4a1 allelic series and the identification of the first two Col4a2 mutant alleles. Genetics 175, 725–736 (2007). 17179069 10.1534/genetics.106.064733 PMC1800636

[b18] Kuo D. S. *et al.* Allelic heterogeneity contributes to variability in ocular dysgenesis, myopathy and brain malformations caused by Col4a1 and Col4a2 mutations. Hum Mol Genet 23, 1709–1722 (2014). 24203695 10.1093/hmg/ddt560 PMC3943517

[b19] Spaide R. F. & Curcio C. A. Anatomical correlates to the bands seen in the outer retina by optical coherence tomography: literature review and model. Retina 31, 1609–1619 (2011). 21844839 10.1097/IAE.0b013e3182247535 PMC3619110

[b20] Labelle-Dumais C. *et al.* COL4A1 mutations cause ocular dysgenesis, neuronal localization defects, and myopathy in mice and Walker-Warburg syndrome in humans. PLoS Genet 7, e1002062 (2011). 21625620 10.1371/journal.pgen.1002062 PMC3098190

[b21] Bai Y. *et al.* Muller cell-derived VEGF is a significant contributor to retinal neovascularization. J Pathol 219, 446–454 (2009). 19768732 10.1002/path.2611

[b22] Iacovelli J. *et al.* Generation of Cre transgenic mice with postnatal RPE-specific ocular expression. Invest Ophthalmol Vis Sci 52, 1378–1383 (2011). 21212186 10.1167/iovs.10-6347 PMC3101664

[b23] Braren R. *et al.* Endothelial FAK is essential for vascular network stability, cell survival, and lamellipodial formation. J Cell Biol 172, 151–162 (2006). 16391003 10.1083/jcb.200506184 PMC2063542

[b24] Thanos A. *et al.* Evidence for baseline retinal pigment epithelium pathology in the Trp1-Cre mouse. Am J Pathol 180, 1917–1927 (2012). 22429967 10.1016/j.ajpath.2012.01.017 PMC3349832

[b25] Hartnett M. E., Weiter J. J., Staurenghi G. & Elsner A. E. Deep retinal vascular anomalous complexes in advanced age-related macular degeneration. Ophthalmology 103, 2042–2053 (1996). 9003338 10.1016/s0161-6420(96)30389-8

[b26] Yannuzzi L. A. *et al.* Retinal angiomatous proliferation in age-related macular degeneration. Retina 21, 416–434 (2001). 11642370 10.1097/00006982-200110000-00003

[b27] Viola F., Massacesi A., Orzalesi N., Ratiglia R. & Staurenghi G. Retinal angiomatous proliferation: natural history and progression of visual loss. Retina 29, 732–739 (2009). 19516115 10.1097/IAE.0b013e3181a395cb

[b28] Sigler E. J. & Calzada J. I. Retinal Angiomatous Proliferation with Chorioretinal Anastomosis in Childhood Coats Disease: A Reappraisal of Macular Fibrosis Using Multimodal Imaging. Retina 35, 537–546 (2015). 25170864 10.1097/IAE.0000000000000341

[b29] Gilmour D. F. Familial exudative vitreoretinopathy and related retinopathies. Eye (Lond) 29, 1–14 (2015). 25323851 10.1038/eye.2014.70 PMC4289842

[b30] Freund K. B. *et al.* Type 3 neovascularization: the expanded spectrum of retinal angiomatous proliferation. Retina 28, 201–211 (2008). 18301024 10.1097/IAE.0b013e3181669504

[b31] Hasegawa E. *et al.* Characterization of a spontaneous retinal neovascular mouse model. PLoS One 9, e106507 (2014). 25188381 10.1371/journal.pone.0106507 PMC4154693

[b32] Hu W. *et al.* Expression of VLDLR in the retina and evolution of subretinal neovascularization in the knockout mouse model’s retinal angiomatous proliferation. Invest Ophthalmol Vis Sci 49, 407–415 (2008). 18172119 10.1167/iovs.07-0870

[b33] Li C. *et al.* Biochemical alterations in the retinas of very low-density lipoprotein receptor knockout mice: an animal model of retinal angiomatous proliferation. Arch Ophthalmol 125, 795–803 (2007). 17562991 10.1001/archopht.125.6.795

[b34] Nagai N. *et al.* Spontaneous CNV in a novel mutant mouse is associated with early VEGF-A-driven angiogenesis and late-stage focal edema, neural cell loss, and dysfunction. Invest Ophthalmol Vis Sci 55, 3709–3719 (2014). 24845632 10.1167/iovs.14-13989 PMC4059080

[b35] Xia C. H., Yablonka-Reuveni Z. & Gong X. LRP5 is required for vascular development in deeper layers of the retina. PLoS One 5, e11676 (2010). 20652025 10.1371/journal.pone.0011676 PMC2907392

[b36] Chen Y., Hu Y., Lu K., Flannery J. G. & Ma J. X. Very low density lipoprotein receptor, a negative regulator of the wnt signaling pathway and choroidal neovascularization. J Biol Chem 282, 34420–34428 (2007). 17890782 10.1074/jbc.M611289200

[b37] Multhaupt H. A. *et al.* Expression of very low density lipoprotein receptor in the vascular wall. Analysis of human tissues by *in situ* hybridization and immunohistochemistry. Am J Pathol 148, 1985–1997 (1996). 8669483 PMC1861641

[b38] Ye X. *et al.* Norrin, frizzled-4, and Lrp5 signaling in endothelial cells controls a genetic program for retinal vascularization. Cell 139, 285–298 (2009). 19837032 10.1016/j.cell.2009.07.047 PMC2779707

[b39] Sudhakar A. *et al.* Human alpha1 type IV collagen NC1 domain exhibits distinct antiangiogenic activity mediated by alpha1beta1 integrin. J Clin Invest 115, 2801–2810 (2005). 16151532 10.1172/JCI24813 PMC1199529

[b40] Rebustini I. T. *et al.* MT2-MMP-dependent release of collagen IV NC1 domains regulates submandibular gland branching morphogenesis. Dev Cell 17, 482–493 (2009). 19853562 10.1016/j.devcel.2009.07.016 PMC2768621

[b41] Knighton D. R. *et al.* Oxygen tension regulates the expression of angiogenesis factor by macrophages. Science 221, 1283–1285 (1983). 6612342 10.1126/science.6612342

[b42] Kelly B. D. *et al.* Cell type-specific regulation of angiogenic growth factor gene expression and induction of angiogenesis in nonischemic tissue by a constitutively active form of hypoxia-inducible factor 1. Circ Res 93, 1074–1081 (2003). 14576200 10.1161/01.RES.0000102937.50486.1B

[b43] Yamakawa M. *et al.* Hypoxia-inducible factor-1 mediates activation of cultured vascular endothelial cells by inducing multiple angiogenic factors. Circ Res 93, 664–673 (2003). 12958144 10.1161/01.RES.0000093984.48643.D7

[b44] Carmeliet P. *et al.* Synergism between vascular endothelial growth factor and placental growth factor contributes to angiogenesis and plasma extravasation in pathological conditions. Nat Med 7, 575–583 (2001). 11329059 10.1038/87904

[b45] Feeney S. A. *et al.* Role of vascular endothelial growth factor and placental growth factors during retinal vascular development and hyaloid regression. Invest Ophthalmol Vis Sci 44, 839–847 (2003). 12556420 10.1167/iovs.02-0040

[b46] Sakurai E., Anand A., Ambati B. K., van Rooijen N. & Ambati J. Macrophage depletion inhibits experimental choroidal neovascularization. Invest Ophthalmol Vis Sci 44, 3578–3585 (2003). 12882810 10.1167/iovs.03-0097

[b47] Nishimura T., Goodnight R., Prendergast R. A. & Ryan S. J. Activated macrophages in experimental subretinal neovascularization. Ophthalmologica 200, 39–44 (1990). 2181360 10.1159/000310075

[b48] Kruegel J., Rubel D. & Gross O. Alport syndrome--insights from basic and clinical research. Nat Rev Nephrol 9, 170–178 (2013). 23165304 10.1038/nrneph.2012.259

[b49] Savige J. *et al.* Retinal basement membrane abnormalities and the retinopathy of Alport syndrome. Invest Ophthalmol Vis Sci 51, 1621–1627 (2010). 19850830 10.1167/iovs.08-3323 PMC2868425

[b50] Vahedi K. & Alamowitch S. Clinical spectrum of type IV collagen (COL4A1) mutations: a novel genetic multisystem disease. Curr Opin Neurol 24, 63–68 (2011). 21157337 10.1097/WCO.0b013e32834232c6

[b51] Shah S. *et al.* Childhood presentation of COL4A1 mutations. Dev Med Child Neurol 54, 569–574 (2012). 22574627 10.1111/j.1469-8749.2011.04198.x

[b52] Kowalczuk L. *et al.* Placental growth factor contributes to micro-vascular abnormalization and blood-retinal barrier breakdown in diabetic retinopathy. PLoS One 6, e17462 (2011). 21408222 10.1371/journal.pone.0017462 PMC3049767

[b53] Zenteno J. C. *et al.* Next generation sequencing uncovers a missense mutation in COL4A1 as the cause of familial retinal arteriolar tortuosity. Graefes Arch Clin Exp Ophthalmol 252, 1789–1794 (2014). 25228067 10.1007/s00417-014-2800-6

[b54] Wang F., Wang J., Liu D. & Su Y. Normalizing genes for real-time polymerase chain reaction in epithelial and nonepithelial cells of mouse small intestine. Anal Biochem 399, 211–217 (2010). 20036209 10.1016/j.ab.2009.12.029

