# Supplementary material for: Col4a1 mutations cause progressive retinal neovascular defects and retinopathy
Source: Sci Rep. 2016 Jan 27;6:18602. doi: 10.1038/srep18602 (PMC4728690; doi:10.1038/srep18602)
Supplement: Supplementary Information [file srep18602-s1.doc]

**Supplemental Files**

***Col4a1* mutation causes progressive retinal neovascular defects and retinopathy**

Marcel V. Alavi^1^, Mao Mao^1^, Bradley T. Pawlikowski^1,2^, Manana Kvezereli^1^,
Jacque L. Duncan^1^, Richard T. Libby^3^, Simon W.M. John^4^ and Douglas B. Gould^1,5^*

^1^ Department of Ophthalmology, School of Medicine, University of California, San Francisco, San Francisco, CA, 94143

^2^ Present address: Department of Molecular, Cellular and Developmental Biology, University of Colorado, Boulder, CO, 80309

^3^ Department of Ophthalmology, University of Rochester, Rochester, NY, 14642

^4^ Howard Hughes Medical Institute and The Jackson Laboratory, Bar Harbor, ME 04609

^5^ Department of Anatomy and Institute for Human Genetics, School of Medicine, University of California, San Francisco, San Francisco, CA, 94143

**Supplemental Materials and Methods**

Primer sequences for quantification of angiogenic regulator gene expression:

Angpt1_F, 5´–CACATAGGGTGCAGCAACCA–3´

Angpt1_R, 5´–CGTCGTGTTCTGGAAGAATGA–3´

Angpt2_F, 5´–CCTCGACTACGACGACTCAGT–3´

Angpt2_R, 5´–TCTGCACCACATTCTGTTGGA–3´

Pdgfb_F, 5´–CATCCGCTCCTTTGATGATCTT–3´

Pdgfb_R, 5´–GTGCTCGGGTCATGTTCAAGT–3´

Pgf_F, 5´–TCTGCTGGGAACAACTCAACA–3´

Pgf_R, 5´–GTGAGACACCTCATCAGGGTAT–3´

Tbp_F, 5´–CAAACCCAGAATTGTTCTCCTT–3´

Tbp_R, 5´–ATGTGGTCTTCCTGAATCCCT–3´

Vegfa_F, 5´–GCACATAGGAGAGATGAGCTTCC–3´

Vegfa_R, 5´–CTCCGCTCTGAACAAGGCT–3´

Vegfb_F, 5´–TGACATCATCCATCCCACTC–3´

Vegfb_R, 5´–CCTTGGCAATGGAGGAAG–3´

Vegfc_F, 5´–GAGGTCAAGGCTTTTGAAGGC–3´

Vegfc_R, 5´–CTGTCCTGGTATTGAGGGTGG–3´

Primer sequences for validation of the conditional *Col4a1^Flex41^* allele:

F1, 5´–CCAGAGGCCACTTGTGTAGC–3´

R1, 5´–AAGACACACGTGATCCACTGGTTC–3´

F2, 5´–TGATAGAAGAGCTGTCGTGGCAG –3´

R2, 5´–CTTCATCCCGTCGAGTGGGTGG–3´

R3, 5´–AGCGCATCGCCTTCTATCGCCTTC–3´

exon_41-F, 5´–TCTTTAAGTGCTGCGTGGTG–3´

exon_41-R, 5´–GGACCACTCCACACAAAGGT–3´

exon_41_RC-F, 5´–TGGCTGCTCTTCAGTTCCCTT–3´

exon_41_RC-R, 5´–CGTCCGAAGAACGGATCCAA–3´.

| **Mutation** | COL4A1 | COL4A2 | COL4A1 | COL4A1 | COL4A1 | COL4A1 | COL4A1 | COL4A1 |
| --- | --- | --- | --- | --- | --- | --- | --- | --- |
|  | G394V | G646D | G658D | G912V | G1038S | G1180D | G1344D | S1582P |
| **Eyes showing retinopathy  (all examined eyes)** | 10  (36) | 7 (30) | 1 (20) | 9 (10) | 5 (10) | 8 (28) | 8 (16) | 1 (4) |
| **Age at clinical assessment** | 1-10 mo | 5-8 mo | 5-12 mo | 8-10 mo | 6-9 mo | 2-9 mo | 3-7 mo | 9-12 mo |

**Supplemental Table S1**

**Supplemental Figures**

**Supplemental Figure S1: All *Col4a1*mutant mouse lines and *Col4a2*mutant mice showed retinopathy.** Fundus examinations of the indicated *Col4a1* mutant mouse lines revealed variable degrees of retinopathy with a more pronounced phenotype for glycine mutations near the carboxy terminal NC1 domain.

**Supplemental Figure S2: Example of a unilateral focal retinal atrophy seen in a *Col4a1^+/+^* mouse.** Fundus of the right eye of a 20-month old *Col4a1^+/+^* mouse **(a)** was without any pathological findings, while the left eye showed focal retinal atrophy **(b)**. Optical coherence tomography (OCT) indicated that all retinal layers were gone, leaving a “retinal hole” in this eye **(c)**. This type of retinal atrophy was seen in 2 of 140 *Col4a1^+/+^* eyes. Bars: 100 µm each.

**Supplemental Figure S3: Example of a unilateral vitreous hemorrhage seen in a *Col4a1^+/Δex41^* mouse.** *Col4a1^+/Δex41^* mouse presented unilateral with an acute dense vitreous hemorrhage **(arrow in a)** that impeded retinal *in vivo* assessment at P45. The vitreous hemorrhage resolved by P90 and funduscopy revealed irregular lesions in this eye **(b)**. OCT uncovered focal adhesions of the posterior vitreous to the retina **(c)**. These findings extend the variety of ocular phenotypes found in *Col4a1^+/Δex41^* mice. Arrows indicate the posterior vitreous. Bars: 100 µm each.

**Supplemental Figure S4: Ectopic immunolabeling for collagen IV demonstrated neovascularization in eyes from *Col4a1^+/Δex41^* mice.** Representative retinal sections of eyes from 6-month old *Col4a1^+/+^* **(a–c)** and *Col4a1^+/Δex41^* **(d–i)** mice demonstrate ectopic collagen IV labeling (green) in retinas from *Col4a1^+/Δex41^* mice indicating the presence of abnormal blood vessels in the outer retina **(e, h)**. A pronounced collagen IV signal at the RPE-photoreceptor complex also indicates fibrosis in *Col4a1^+/Δex41^* mice **(h)**. These findings clearly demonstrate neovascularization in *Col4a1^+/Δex41^* mice associated with retinal lesions. a, d, g are DIC images and DAPI nuclear staining is blue. Bar: 50 µm.

**Supplemental Figure S5: Electroretinography (ERG) did not reveal differences in retinal function between *Col4a1^+/+^* and *Col4a1^+/Δex41^* mice.**

We compared ERGs of *Col4a1^+/+^* and *Col4a1^+/Δex41^* mice over a range of light intensities. The scotopic a- and b-wave amplitudes **(a)** and b-wave implicit times **(b)** were indistinguishable between *Col4a1^+/Δex41^* and *Col4a1^+/+^* mice. A-wave implicit times tended to be lower in *Col4a1^+/Δex41^* mice but were not significantly different **(c)**. The absence of major ERG defects suggests that the focal lesions are not extensive enough to compromise the recording of pan-retinal function on full field ERG (n≥20 for each group).

**Supplemental Figure S6: Design and validation of the conditional *Col4a1^+/Flex41^* mouse line.** The conditional *Col4a1^Flex41^* allele was designed with *LoxP* sites flanking exon 41 **(a, b)**, which – upon Cre-recombinase expression – will resemble the splice-site mutation of *Col4a1^+/Δex41^* mice. Pups that carry the downstream and upstream *LoxP* sites were identified by PCR using primers F1 and R1 **(c)** and primers F2 and R2, respectively **(d)**. Crossing *Best1^Cre^* mice to tomato reporter mice **(e,** *Tom^fl^***)** showed a uniform signal in the retina of *Best1^Cre^* ;*Tom^fl^* mice **(f)** consistent with expression of the Cre recombinase in RPE cells. To confirm that *Tie2^Cre^ ;Col4a1^+/Flex41^* mice express mutant collagen in vascular endothelial cells, we also crossed in the tomato reporter transgene and examined animals by fluorescence ophthalmoscopy. A *Tie2^Cre^ ;Col4a1^+/Flex41^*;*Tom^fl^* mouse showed a small defined lesion in funduscopy **(g)** and the vasculature was fluorescently labeled by the tomato reporter **(h).** Additionally, *Col4a1* exon 41 was readily amplified from genomic DNA from tail biopsies, retina and RPE from *Col4a1^+/Flex41^* and *Best1^Cre^*;*Col4a1^+/Flex41^* mice **(i),** however, RT-PCR with recombination specific primers confirmed that *Col4a1* exon 41 was excised in RPE only in this strain **(j)**. Taken together, conditional *Best1^Cre^ ;Col4a1^+/Flex41^* mice and *Tie2^Cre^ ;Col4a1^+/Flex41^* mice express mutant collagen in RPE and vascular endothelial cells, respectively.

**Supplemental Figure S7: Funduscopy and image guided OCT showed anastomosis connecting a retinal artery with the choroid in a *Col4a1^+/Δex41^* mouse.** Funduscopy showed a defined retinal lesion in a 1 year-old *Col4a1^+/Δex41^* mouse **(a)**. At higher magnifications, the vessel appears to originate from a normally branched vein. **(b, arrows)**. OCT confirmed the anastomosis of a retinal artery and the choroid **(c, arrows)**. This suggests that the superficial vessels, and not the deep plexus, contribute to neovascularization. Bars: 100 µm each.

**Supplemental 3D-reconstruction V1: Focal adhesions of the posterior vitreous to the retina in a *Col4a1^+/Δex41^* mouse.** OCT 3D-model of the eye shown in Supplemental Figure S2 showing the focal adhesion of the posterior vitreous to the retina.

**Supplemental 3D-reconstruction V2: Anastomosis connecting a retinal artery with the choroid in a *Col4a1^+/Δex41^* mouse.** OCT 3D-model showing an anastomosis of a retinal artery and the choroid in a 1 year-old *Col4a1^+/Δex41^* mouse. (Blood vessels have been manually colored.)

**Supplemental 3D-reconstruction V3: Retinal artery approaching the photoreceptor layers in a *Col4a1^+/Δex41^* mouse.** OCT 3D-model of a retinal artery diving into the retinal outer layers towards the photoreceptor/choroid in a six week-old *Col4a1^+/Δex41^* mouse.

**Supplemental 3D-reconstruction V4: Abnormal blood vessel in a *Tie2^Cre^ ;Col4a1^+/Flex41^* mouse.** OCT 3D-model of a blood vessel in the retinal outer layers in a three week-old *Tie2^Cre^ ;Col4a1^+/Flex41^* mouse.
